# Supplementary material for: Hydrogen Nanometrology in Advanced Carbon Nanomaterial Electrodes
Source: Nanomaterials (Basel). 2021 Apr 22;11(5):1079. doi: 10.3390/nano11051079 (PMC8143510; doi:10.3390/nano11051079)
Supplement: Supplementary file 1 [file nanomaterials-11-01079-s001.zip › nanomaterials-1162774-supplementary.pdf]

Supplementary Materials

Hydrogen Nanometrology in Advanced Carbon Nanomaterial Electrodes

Rui Lobo [1,2,*], Noe Alvarez [3] and Vesselin Shanov [4]

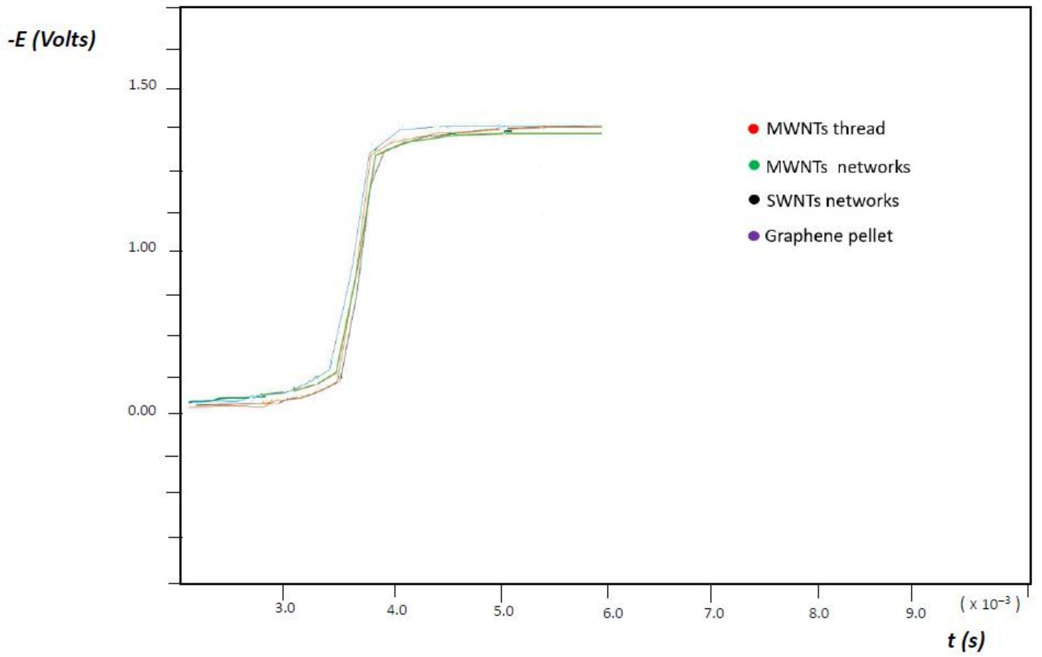

Figure S1. Galvanostatic charge of hydrogen in the four studied nanostructured carbon cathodes, in NaOH 1 M solution. Each of the four systems is first allowed to equilibrate for one hour, and then a negative polarization of 1 V (and current of 133 mA) is applied during one more hour, prior of being probed by MB-TDS.
